# Inhibition of LPS-induced airway neutrophilic inflammation in healthy volunteers with an oral CXCR2 antagonist

**DOI:** 10.1186/1465-9921-14-137

**Published:** 2013-12-16

**Authors:** Brian R Leaker, Peter J Barnes, Brian O’Connor

**Affiliations:** 1 Respiratory Clinical Trials Ltd, 20 Queen Anne Street, London W1G 8HU, UK; 2 National Heart & Lung Institute, Imperial College, London SW3 6LY, UK

**Keywords:** Neutrophil, Chemokine receptor, CXCL1, COPD, Severe asthma, Cystic fibrosis, Endotoxin

## Abstract

**Background:**

Inhaled lipopolysaccharide (LPS) induces a dose-dependent, acute neutrophilic response in the airways of healthy volunteers that can be quantified in induced sputum. Chemokines, such as CXCL1 and CXCL8, play an important role in neutrophilic inflammation in the lung through the activation of CXCR2 and small molecule antagonists of these receptors have now been developed. We investigated the effect of AZD8309, a CXCR2 antagonist, compared with placebo on LPS-induced inflammation measured in sputum of healthy volunteers.

**Methods:**

Twenty healthy subjects were randomized in a double-blind placebo-controlled, cross-over study. AZD8309 (300 mg) or placebo was dosed twice daily orally for 3 days prior to challenge with inhaled LPS and induced sputum was collected 6 h later.

**Results:**

Treatment with AZD8309 showed a mean 77% reduction in total sputum cells (p < 0.001) and 79% reduction in sputum neutrophils (p < 0.05) compared with placebo after LPS challenge. There was also a reduction in neutrophil elastase activity (p < 0.05) and CXCL1 (p < 0.05) and trends for reductions in sputum macrophages (47%), leukotriene B_4_ (39%) and CXCL8 (52%).

**Conclusions:**

AZD8309 inhibited LPS-induced inflammation measured in induced sputum of normal volunteers, indicating that this treatment may be useful in the treatment of neutrophilic diseases of the airways, such as COPD, severe asthma and cystic fibrosis.

**Trial registration:**

NCT00860821.

## Background

Several chronic inflammatory diseases of the lung, including chronic obstructive pulmonary disease (COPD), cystic fibrosis (CF), severe asthma, acute respiratory distress syndrome and bronchiolitis obliterans syndrome, are characterised by a predominantly neutrophilic pattern of inflammation, which is relatively resistant to the anti-inflammatory effects of corticosteroids, making them difficult to manage in clinical practice. Several new anti-inflammatory treatments currently in development for these diseases such as phosphodiesterase(PDE)-4 and p38 mitogen activated protein (MAP) kinase inhibitors, target neutrophilic inflammation, but their clinical development has been hampered by dose-limiting side effects after oral administration [1,2]. Neutrophilic inflammation in the lungs is driven by the release of chemotactic factors secreted by structural cells within the lung, such as epithelial and airway smooth muscle cells, and by resident inflammatory cells, such as macrophages and recruited neutrophils. The major neutrophil chemoattractants are CXC chemokines, including CXCL1 (GRO-α), CXCL5 (ENA78) and CXCL8 (interleukin-8), all of which are increased in concentration in bronchoalveolar lavage of patients with COPD, severe asthma and CF [3-9]. These neutrophil chemokines activate a common chemokine receptor CXCR2, which is expressed on the surface of neutrophils. The tripeptide PGP, which is generated from extracellular matrix proteins through the action of matrix metalloproteinases, is also a potent neutrophil chemoattractant (matrikine) that activates CXCR2 [10,11]. These chemokines also attract circulating monocytes, which differentiate within the lung to macrophages that are thought to drive neutrophilic inflammation [12]. This suggests that blocking CXCR2 would be an attractive therapeutic approach to the treatment of neutrophilic inflammation, since small molecule inhibitors to these G-protein receptors have now been developed [13,14].

Several small molecule CXCR2 antagonists have now been developed for oral administration [13]. Oral administration of the CXCR2 antagonists navarixin (SCH527123) and SB-656933 have previously been shown to inhibit the increased neutrophils in induced sputum as a result of ozone challenge in normal volunteers [15,16]. Ozone challenge is difficult as it involves prolonged administration of ozone in specially designed exposure chambers and the need for subjects to exercise. Inhaled lipopolysaccharide (LPS, endotoxin) is well established as an inducer of neutrophilic inflammation in normal volunteers and is easier to administer than ozone [17-20]. We therefore investigated whether inhaled LPS challenge could be used to assess the anti-neutrophilic effect of a CXCR2 antagonist AZD8309 in normal volunteers.

Studies in animals have shown that neutrophilic inflammation induced by LPS is attenuated in CXCR2-deficient mice [21] and small molecule CXCR2 antagonists inhibit pulmonary neutrophilic inflammation induced by inhaled LPS in several animal species [22]. AZD8309 is a potent small molecule reversible CXCR2 antagonist. Recently oral administration of ADZ8309 has been shown to inhibit neutrophilia induced by endotoxin instillation into the nose of normal subjects [23]. In the present double-blind placebo controlled cross-over study we investigated the effect of orally administered ADZ8309 on pulmonary inflammation induced by inhaled LPS in healthy subjects, by measuring inflammatory cells and mediators in induced sputum.

## Methods

### Study design

**Figure 1:**
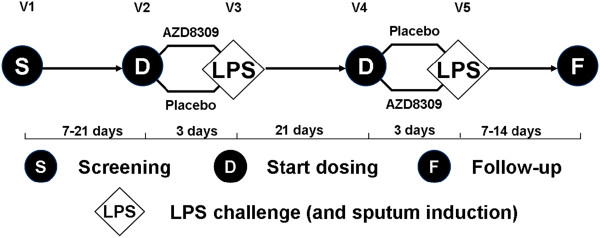
**Study design.** After a screening visit (visit 1) treatment period 1 was comprised of visits 2 and 3 and treatment period 2 was comprised of visits 4 and 5. Eligible randomized subjects returned to the clinic (visit 2) 7–21 days following visit 1 and were dosed with either 300 mg AZD8309 or placebo at 09:00 hours and were discharged with instructions for continued dosing at home. Subjects returned for LPS challenge the following morning (visit 3). Six hours post-completion of the LPS inhalation, subjects produced an induced sputum sample. There was a washout period between visits 3 and 4 of at least 21 days. Treatment period 2 was structured the same as treatment period 1.

We used a double-blind, placebo-controlled two-way crossover design in which healthy adult subjects were recruited in accordance with the Good Clinical Practice principles outlined by the Declaration of Helsinki. The study was powered to detect a 50% reduction in sputum neutrophil counts, with a 80% power at 5% level. 16 subjects were deemed sufficient to complete the study in a cross-over design to detect a difference between active drug versus placebo. The study was approved by Brent Medical Ethics Committee (London) and all subjects provided written informed consent prior to any study related procedures. Figure 1 shows the design and sequence of the study as a flow chart. The subjects were non-smokers or ex-smokers for at least 12 months with a pack year history <10, FEV_1_ ≥ 80% predicted normal and FEV_1_/FVC >70%, normal airway responsiveness to inhaled methacholine with a PC_20_ ≥ 16 mg/mL and able to produce a minimum of 200 μL sputum volume after sputum induction at screening.

Demographic measurements were recorded on the first clinical visit (Visit 1 screening). At Visit 2, subjects were randomized to treatment and dosed with either an oral suspension of 300 mg AZD8309 or placebo. A second dose was taken approximately 12 hours later, and a similar dosing regimen was repeated the following day. A fifth dose was taken at the clinic on the morning of day 3 and subjects were challenged with inhaled LPS one hour later. Six hours post-LPS inhalation, hypertonic saline by aerosol was administered to produce an induced sputum sample. A sixth and final dose was administered in the clinic 12 hours after the morning dose to maintain plasma levels.

Blood samples (collected into EDTA) were collected on day 1 prior to randomization to treatment and at 7 hours post dosing, on the morning of day 3 prior dosing and at 6 and 24 hours after LPS challenge for assessment of circulating leukocytes and analysis of AZD8309 concentrations. A washout period of at least 21 days was mandatory before subjects returned to begin dosing with the second treatment period, which was structured as the previous visit. A follow-up visit took place after completion of the final treatment and comprised a final physical examination and a laboratory screen.

A dose of 300 mg AZD8309 was used as this dose resulted in maintaining plasma concentrations of AZD8309 at approximately 3 times the IC_50_ for the CXCR2 receptor at 12 hour post dosing. The vehicle (and placebo treatment) was hydroxypropyl-methylcellulose, meglumine, hydroxypropyl-β-cyclodextrin in water.

### LPS challenge

1 mg lyophilized LPS (*E. coli* 026:B6, Sigma Chemical Company, Poole, UK) was used within 2 hours of reconstitution in sterile, isotonic saline. The subject inhaled 5 breaths of 0.50 mg/mL LPS (30 μg) from a breath-activated Mefar MB3 dosimeter (12 μL per actuation) as previously described [20].

#### Sputum induction and processing

Subjects were given increasing concentrations of hypertonic saline (3%, 4% and 5%) for 5 minute periods using a nebulizer (Medix Ltd, UK). Sputum was collected into a 50 mL universal container for each 5 min period and processed within 120 min of collection. Sputum was processed using dithiothreitol (DTT) at final concentration of 0.1% in PBS according to the published protocol [18,19]. The resulting supernatant was stored at −70°C until analysis.

### Induced sputum analysis

Cell pellets were resuspended in 1–5 mL of PBSA (phosphate buffered saline plus 0.1% bovine serum albumin). Viability assessment and total cell count were performed by the use of trypan blue exclusion staining and a hemocytometer. Samples were diluted in PBSA to give 2 × 10^5^ viable non-squamous cells per mL and centrifuged onto cytospin slides for 3 minutes at 450 rpm. Differential counts were expressed as percentage of total cell counts from Diff-Quik stained cytospin samples (n = 400).

Sputum supernatant samples were analyzed for CXCL1, CXCL8 and leukotriene (LT) B_4_ using commercially available ELISA kits, according to the manufacturer’s guidelines (R & D; GE Healthcare, UK). Neutrophil elastase (NE) activity was determined by an end-point read kinetic assay run in a 96-well plate format. Samples were incubated with N-methoxysuccinyl-Ala-Ala-Pro-Val-7-amino-4-methylcoumarin, (Calbiochem, Nottingham UK), which is cleaved by NE yielding a fluorescent product, 7-amino-methyl-coumarin. Results were reported as fluorescence units and there was no lower limit of quantification applied.

### Statistical analysis

The primary efficacy variable was the neutrophil count in sputum. This was analyzed using a multiplicative 2-period crossover ANOVA: the logarithm of the count was modelled additively with factors treatment, period and patient. The exponential log of the mean treatment difference and its confidence interval was used to compare the ratio of geometric means of the count, for AZD8309 to placebo. The treatment p-value was also computed non-parametrically using Wilcoxon test (the treatment difference was compared between the two randomization sequences). Other sputum variables (macrophage count, relative cell counts as well as immunological mediators) were analyzed in the same way.

## Results

### Subject demographics

Twenty subjects were randomized to treatment at Visit 2 and 16 completed the study. Of the 20 patients randomized all were males, with a mean age of 26 (range 19–44) years. Sixteen were Caucasian, 3 were black and 1 was Asian. The demographic and baseline characteristics of study subjects are summarized in Table 1. Four subjects failed to complete the study: 1 subject withdrew consent, 1 patient in the placebo group withdrew from the study due to a migraine, 1 subject was withdrawn because they had screened for another clinical trial at another unit, and another subject was withdrawn because they were found to have been over-volunteering for clinical studies. Of the four subjects who withdrew from the study, two of them received the LPS challenge (both on placebo treatment) and all of them received at least one dose of study drug.

**Table 1:** Demographic and baseline characteristics of subjects

***Parameter***	***All subjects (n = 20)***
**Sex: Male**	20
**Age (yr)**	26.0 (range 19–44)
**Race:**	
Caucasian	16
Black	3
Other	1
BMI (kg/m^2^)	24.6 (range 20–30)
**Smoking status:**	
Never	17
Previous	3

Pharmacokinetic analysis of blood samples taken post dosing showed that all subjects achieved plasma concentrations of AZD8309 above the concentration for 3 × IC_50_ to antagonize the CXCR2 receptor.

In one subject, there was insufficient sputum pellet to generate a cytospin for a differential count and on three occasions there was insufficient sputum supernatant to be able to do a paired analysis of CXCL1 levels.

### Sputum inflammatory cells

Sputum induction and collection were carried out at Visit 1 to assess whether the subject could produce sufficient volumes, and then again in each treatment period 6 hours post-LPS challenge. Table 2 shows the geometric means of the total cell counts for all leukocytes, neutrophils and macrophages in these sputum samples. There were only small numbers of eosinophils and lymphocytes (which were not analyzed further), while most of the cells were macrophages or neutrophils (Table 2).

**Table 2:** Cell count numbers in sputum after LPS challenge in each treatment group

**Cell type (x10**^**6**^**/g)**	**N**	**Geometric mean**				**P**	**P#**
		**AZD8309**	**CV (%)**	**Placebo**	**CV (%)**		
Total cells	16	5.5	804	24	252	<0.001	0.003
Neutrophils	15	3.7	1595	17	252	0.027	0.028
Macrophages	15	1.9	354	3.6	523	0.15	0.11

P = value by ANOVA; P# = value by Wilcoxon test.

There is only n = 15 for the neutrophil and macrophage numbers because it was not possible to get a cell differential count from one of the sputum cytopsin preparations.

**Figure 2:**
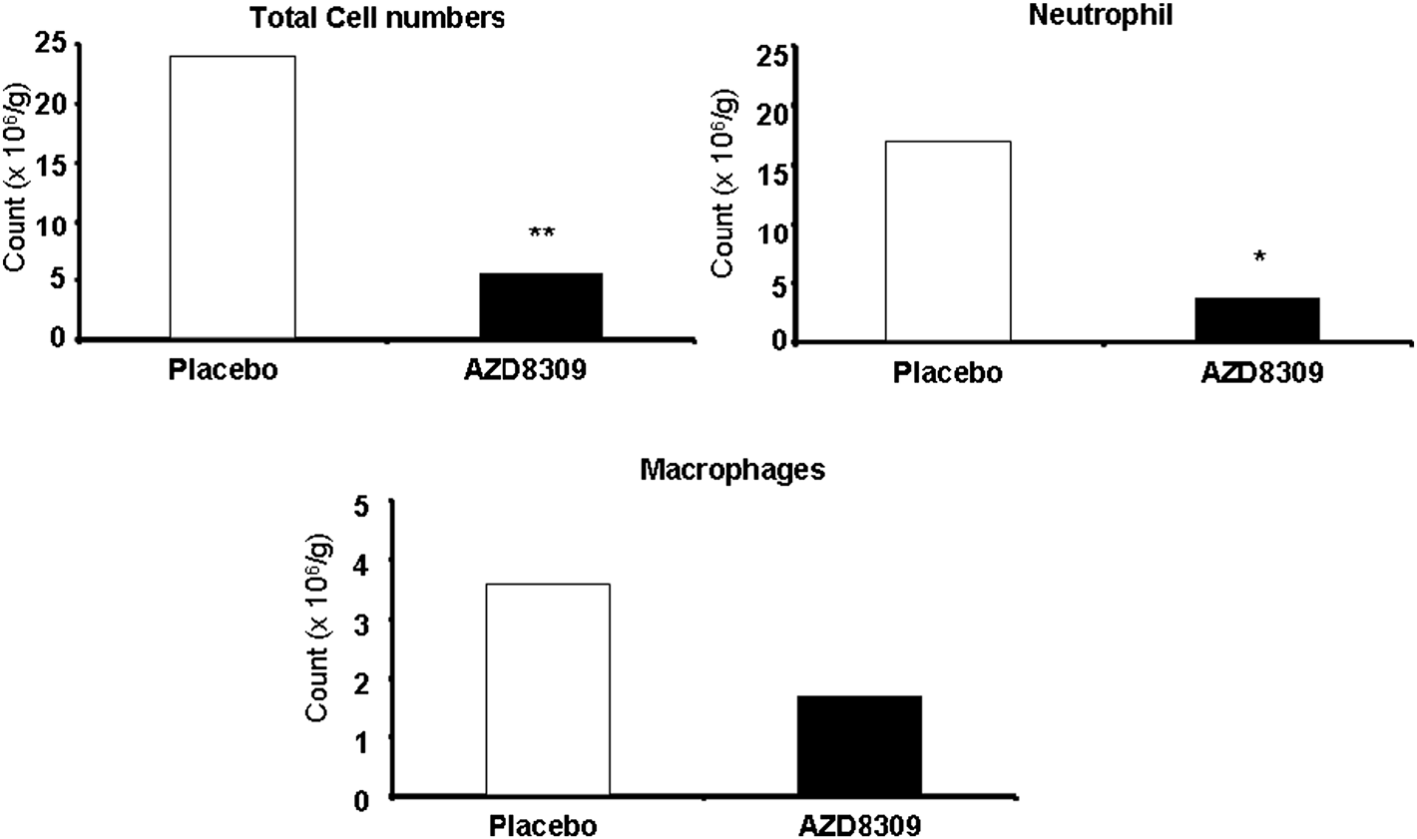
**Effect of AZD8309 on cell counts in sputum post LPS challenge.** Total cell counts (top left plot) were performed by trypan blue exclusion with a hemocytometer. Differential cell counts for neutrophils (top right plot) and macrophages (bottom centre plot) were performed with cytospin preparations. Bars show the mean values of 15–16 patients. Open bars represent placebo and solid bars AZD8309. Results are expressed as geometric mean. CVs are shown in Table 2. Statistics by ANOVA, ** = P < 0.001 and * = P < 0.05.

Figure 2 shows the effects of treatment on total leukocytes, neutrophils and macrophages in sputum. The total leukocyte count was reduced by a mean of 77% (*P* < 0.001) after AZD8309 therapy compared to placebo levels. There was a mean 79% decrease in the neutrophil numbers after treatment with AZD8309 compared with placebo (*P* = 0.027). Macrophages were reduced by approximately 50% compared to placebo but this did not reach statistical significance.

### Inflammatory mediators

**Figure 3:**
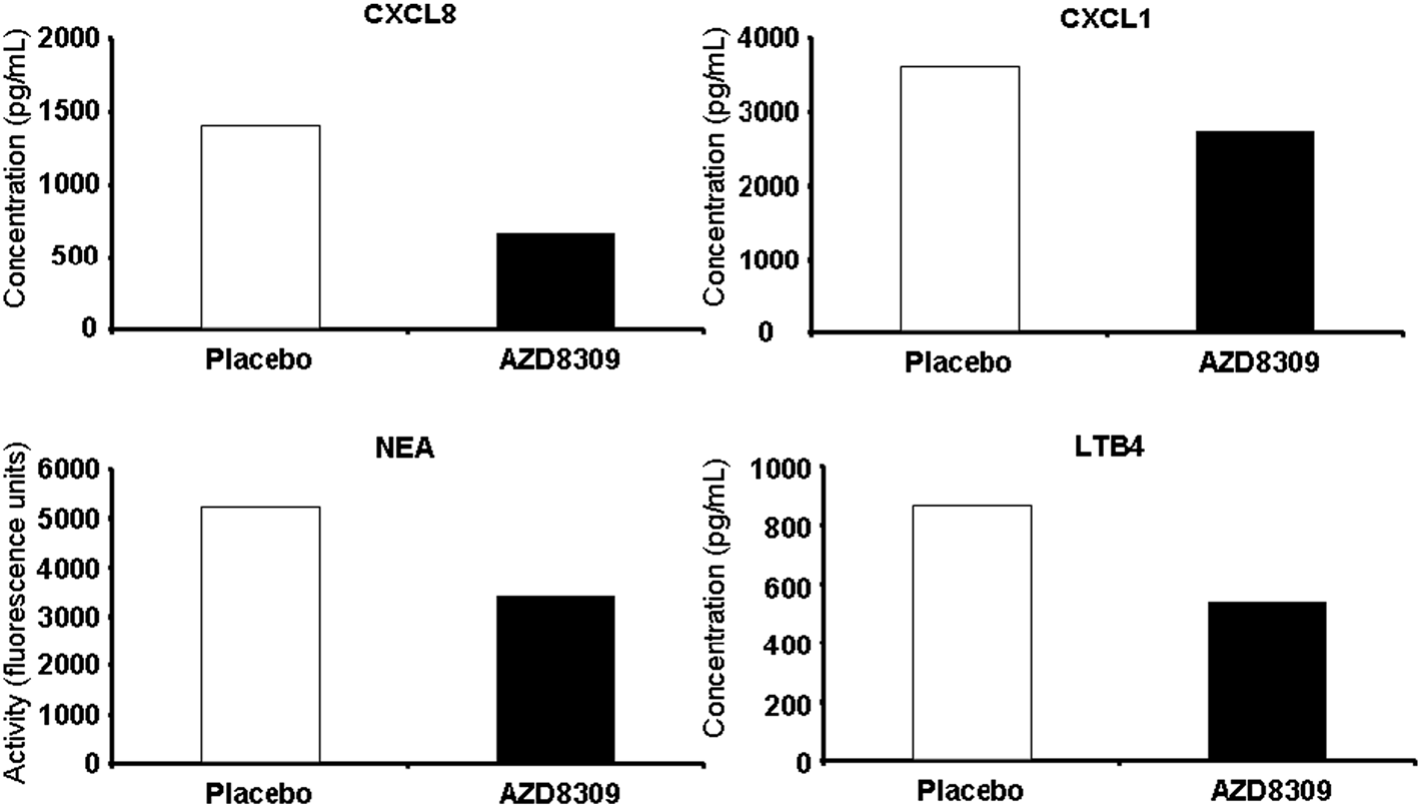
**Effect of AZ8309 on mediator concentrations in sputum supernatant.** Sputum samples were collected 6 hours post challenge with LPS. There was a reduction in concentrations of CXCL8 (top left plot), CXCL1 (top right plot), neutrophil elastase activity (NEA) (bottom left plot) and leukotriene B_4_ (LTB_4_) (Bottom right plot); mean values of 13–16 patients are shown. Open bars represent placebo and solid bars AZD8309. Results are expressed as geometric mean. CVs are shown in Table 3. Statistics by ANOVA, * = P < 0.05.

Figure 3 and Table 3 show the treatment effects on inflammatory mediators in induced sputum after LPS challenge. All were reduced in concentration after treatment with AZD8309 compared to placebo. There was a mean 52% reduction in CXCL8 after AZD8309 group compared to placebo, although this did not reach statistical significance (*P* = 0.1), 35% reduction in NE (*P* = 0.012), 25% lower concentrations of CXCL1 (*P* = 0.044) and 39% lower concentrations of LTB_4_ (p = 0.075). Significance values were similar for ANOVA and Wilcoxon tests, although a non-parametric test showed borderline significance effect on CXCL8 rather than a non-significant result.

**Table 3:** Soluble mediator concentrations in sputum supernatants after LPS challenge in each treatment group

**Mediator**	**N**	**Arithmetic mean**				**Geometric mean**				**P**	**P#**
		**AZD8309**	**S.D.**	**Placebo**	**S.D.**	**AZD8309**	**CV (%)**	**Placebo**	**CV (%)**		
CXCL8 (pg/mL)	16	1373.8	2076.3	2708.2	2880.9	668	251	1404	262	0.1	0.046
CXCL1 (pg/mL)	13	3700.7	5027.0	3934.7	3453.5	2722	75	3622	73	0.044	0.024
LTB_4_ (ng/mL)	16	0.676	0.530	1.181	0.934	0.54	78	0.87	103	0.075	0.14
NEA (fluor units)	16	3438.8	362.3	6419.4	5793.8	3422	10	5227	66	0.012	0.005

S.D. = standard deviation. P = value by ANOVA; P# = value by Wilcoxon test.

LTB_4_: leukotriene B_4_; NEA: neutrophil elastase activity.

#### Circulating blood leukocytes and mediators

**Figure 4:**
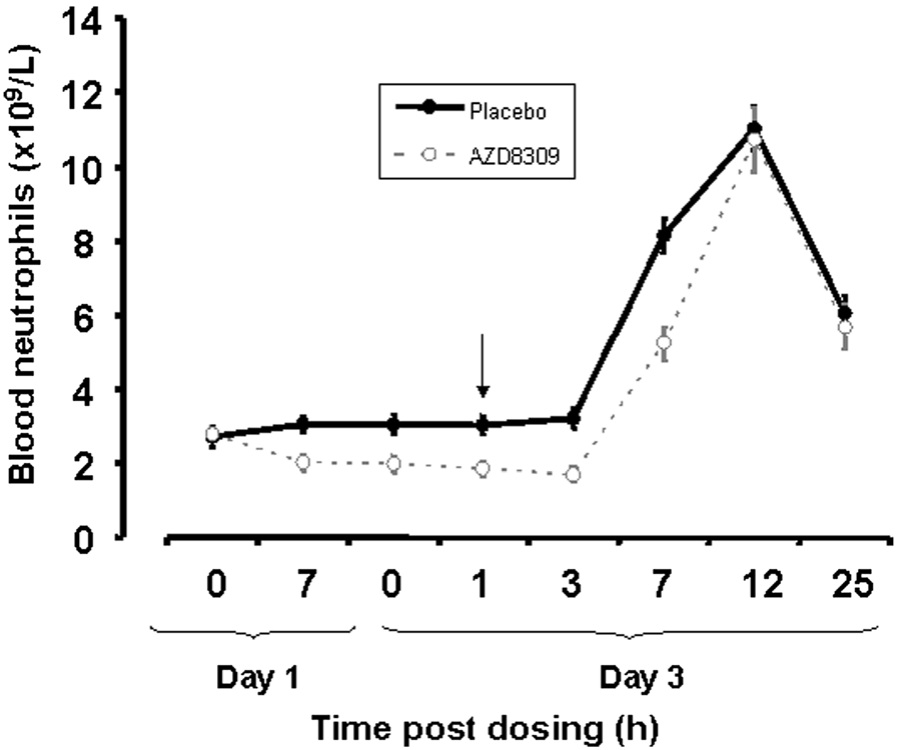
**Effect of AZD8309 on circulating blood neutrophils pre and post challenge with lipopolysaccharide.** Blood neutrophils numbers were measured at predose (t = 0) and at 7 hours post dosing on day 1 and at trough (t = 0) and at 1, 3, 7, 12 and 25 hours post dosing on day 3. The solid black circles show the effects on placebo treatment and the open grey circles show effects after treatment with AZD8309. The arrow indicates the point at which subjects were challenged with LPS on day 3. Each point is presented as mean with s.e.m.

Neutrophil counts in blood were observed to be lower (on average 29%) when subjects received AZD8309 compared to placebo at 7 hours after dosing on day 1 (mean values: 2.71 × 10^9^/L (± 1.15 S.D.) at 0 hours and 3.04 ×10^9^/L (± 0.85 S.D.) at 7 hours on placebo versus 2.77 × 10^9^/L (± 0.80 S.D.) at 0 hours and 2.03 × 10^9^/L (± 0.89 S.D.) at 7 hours on AZD8309). However, on challenge with LPS there was no statistically significant difference between treatment with AZD8309 compared to placebo on blood neutrophil numbers at 6 hours post challenge (8.15 × 10^9^/L (± 1.95 S.D.) on placebo; 5.23 × 10^9^/L (± 1.89 S.D.) on AZD8309, see Figure 4). Blood neutrophil counts had returned to baseline values by follow-up (Data not shown).

C-reactive protein (CRP) and IL-6 levels were also measured in blood at 0, 2, 6 and 25 hours post LPS challenge. In the placebo-treated group, IL-6 levels were highest at 6 hours after LPS challenge (1.20 ± 0.84 SD pg/mL) at 0 hours rising to 34.44 (± 25.94 SD) pg/mL and CRP levels were highest at 24 hours post LPS challenge (0.32 ± 0.03 SD mg/l) at 0 hours rising to 6.45 (± 0.27 SD mg/l) at 24 hours). There was no effect of AZD8309 on blood levels of either IL-6 or CRP.

### Lung function

**Figure 5:**
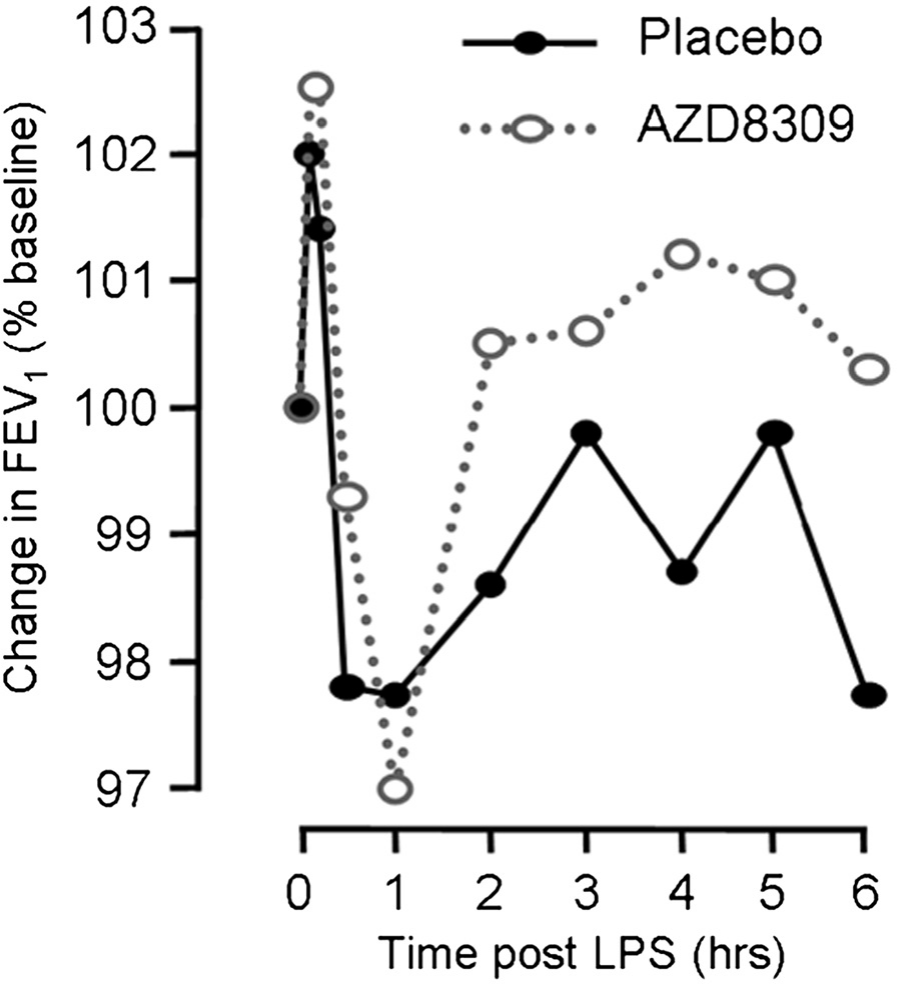
**Effect of AZD8309 on FEV**_**1 **_**after inhaled lipopolysaccharide (LPS).** There was a similar peak fall in FEV_1_ at 1 h after challenge, then a more rapid recovery after AZD8309 compared to placebo, with a significant reduction in area under the curve (P < 0.05). The average values for the coefficient of variances were 14.4% and 15% for AZD8309 and placebo respectively. Mean values of 16 patients are shown.

Spirometry was measured at baseline (1 hour before LPS inhalation) and at 5, 10, 30 min and 1, 2, 3, 4, 5, and 6 hours after inhaled LPS. FEV_1_ changes within the first hour post-LPS challenge were similar for placebo and AZD8309, but there appeared to be a more rapid recovery towards baseline in the subsequent time. Whilst the difference in effect was small it did reach statistical significance; the average FEV_1_ over the 6 hours post LPS challenge was 4.28 L versus 4.23 L (*P* < 0.05) for AZD8309 versus placebo treatment respectively. A time course of FEV_1_ changes for each treatment group is shown in Figure 5.

### Adverse effects

A table of the adverse events after first dose of treatment are shown in Table 4. All adverse effects have been counted once in the period in which they commenced. In general adverse effects were balanced across the treatments. The most frequently reported adverse effects were pyrexia and headache. A total of 44 adverse events were of mild intensity and 3 (all on placebo) were of moderate intensity. There were no serious adverse events in this study.

**Table 4:** Summary of most common adverse events (AE) after dosing in each treatment limb

	***AZD8309 (n = 18)***	***Placebo (n = 19)***
Number of subjects with DAE	0	1 (5%)
Total AEs	19	28
Number of subjects with AEs (%)	14 (61%)	10 (53%)
AEs by preferred term	n = 18	n = 18
pyrexia	5 (28%)	3 (17%)
headache	2 (11%)	4 (22%)
dizziness	0	3 (17%)
nasal congestion	2 (11%)	3 (17%)
diarrhea	3 (17%)	1 (6%)
rhinitis	0	2 (11%)
pharyngolaryngeal pain	0	2 (11%)

The AEs listed are for all subjects who received at least one dose of study drug and occurred in more than one subject. DAE = Discontinuation due to adverse event.

One subject on placebo treatment was discontinued from the study drug due to adverse events. On Day 3 of treatment the subject experienced a migraine (moderate intensity), nausea (mild intensity) and postural dizziness (mild intensity). The subject discontinued treatment the same day.

An increase in body temperature was seen from a mean of 35.9°C pre-dose to 36.9°C at 8 h post-LPS challenge. This had dropped back to 36.3°C at 24 h post-LPS challenge. There were no differences between AZD8309 and placebo in these findings.

There were no differences between the treatment groups in heart rate, systolic or diastolic blood pressure, and no findings of clinical concern in 12-lead ECG.

There were no changes in any mean laboratory values over time, with the exception of leukocytes and neutrophils where decreases of approximately 1×10^9^/L during the AZD8309 treatment period were seen for both measures. Both parameters returned to baseline values by the follow-up visit.

## Discussion

In the present study, the *in vivo* pharmacology of a potent CXCR2 antagonist AZD8309 was evaluated in healthy human subjects using inhalation of LPS, a method which closely replicates key components of the inflammatory response associated with COPD, severe asthma and CF. The main findings of this study are that following LPS challenge, AZD8309 markedly reduced total leukocyte numbers and neutrophil numbers in sputum and reduced sputum concentrations of CXCL1, CXCL8, LTB_4_ and NE. Furthermore, there were no serious adverse events to an LPS challenge of 30 μg or dosing with study drug, indicating that this is a useful and convenient way to initially evaluate the effect of anti-neutrophilic drugs, such as CXCR2 antagonists. Short-term treatment with AZD8309 300 mg b.i.d. was well tolerated with no findings of clinical concern. A potentially interesting additional finding was the observation that AZD8309 administration significantly increased the recovery in FEV_1_ after the small bronchoconstrictor effect of LPS challenge.

Our primary outcome variable of sputum neutrophils revealed approximately 80% reduction in subjects receiving AZD8309 compared to placebo. The increase in neutrophils in sputum obtained in the placebo group was as expected, based on dose–response data previously published [17,20] with a similar increase in inflammatory mediators and markers of neutrophil activation [18,19,24].

The result of the primary variable, together with the fact that sputum biomarkers (CXCL1, CXCL8, NE and LTB_4_) that are markers of neutrophil activation, were observed to be lower in subjects receiving AZD8309, suggests that these effects are mediated through CXCR2. The effect on inflammatory mediators may not mediated directly through CXCR2 but reflect the reduction in numbers of activated neutrophils in the airway, since these cells release CXCL8, LTB_4_ and NE. Overall, the results obtained in this study clearly show that AZD8309 has a positive effect in attenuating the inflammatory response induced by LPS inhalation, and the fact that all subjects achieved trough plasma concentrations of AZD8309 above the pharmacologically active 3 × IC_50_ concentration required to antagonize the CXCR2 receptor for the duration of the study gives confidence that the effects seen were mediated via the expected pharmacological mechanism.

AZD8309 also antagonises the CCR2b receptor but it is 10-fold less potent than at CXCR2 so that even at maximal plasma concentrations achieved in our study there would be little if any antagonism of CCR2b. CCR2 are involved in the recruitment of monocytes form the blood so may be contributory to the increased numbers of macrophages in COPD patients [25]. The reduction in sputum macrophages is more likely explained by an inhibitory effect on CXCR2 which are expressed on circulating monocytes. Our findings are similar to the reported effects of two other CXCR2 antagonists on ozone challenge in normal subjects [15,16]. A preliminary study with the CXCR2 antagonist, navarixin (MK7123, SCH 527123) in patients with COPD showed a reduction in sputum neutrophil counts (by approximately 50%) but no significant change in FEV_1_ after three months of treatment [26]. However in a further study with 6 months dosing, a significant improvement in FEV_1_ of up to 168 ml was demonstrated compared with placebo treated patients [27]. The improvement in FEV_1_ was greatest in a sub-group of patients who continued to smoke and these patients also demonstrated an increased time to their first moderate/severe exacerbation. A reduction in sputum neutrophil counts was also shown in a different subset of these patients. The study also demonstrated a significant dose-related reduction in systemic neutrophil counts compared with placebo although no difference in infection rates were noted compared with the placebo group (see below). Further Nair *et al.* studied SCH527123 [28]. In patients with severe asthma and after four weeks dosing found a 36% reduction in sputum neutrophil percentages; but no significant differences in FEV_1_, IL-8 release or neutrophil elastase release.

Treatment with AZD8309 also showed a reduction in circulating neutrophil numbers. This effect has also been observed in the Phase 1 studies with AZD8309 (unpublished observations) and with navarixin in normal subjects and in COPD patients [15,25]. In the Phase 1 studies the effect on circulating neutrophils was transient and reversible on stopping dosing. Similarly, in this study circulating neutrophil numbers returned to pre-dosing levels as shown in the follow-up visits after stopping treatment with AZD8309. In the present study we were also able to investigate the effect of AZD8309 on blood neutrophil numbers after a pro-inflammatory insult using LPS challenge that leads to mobilization of neutrophils from the bone marrow and a transient increase in circulating neutrophil numbers. AZD8309 had no effect on the observed increase in blood neutrophils after LPS challenge suggesting that neutrophils can be mobilized from the bone marrow regardless of treatment with a CXCR2 antagonist, and demonstrating that this is not a compound that causes bone marrow toxicity and resulting neutropenia. These observations suggest that the apparent reduction in circulating neutrophils produced by CXCR2 antagonists is caused by margination of neutrophils in the pulmonary circulation which can be rapidly mobilised to the systemic circulation.

AZD8309 300 mg bid was well-tolerated and adverse events were similar between treatment and placebo groups. There were no findings of clinical concern, and no indications that AZD8309 adversely affected lung function. The transient increase in body temperature observed in both treatment limbs of the study during the first 8 hours following LPS challenge has previously been reported with previous LPS challenge studies [17-20,24].

CXCR2 is expressed not only on circulating leukocytes but also on smooth muscle, submucosal glands and the central nervous system, so side effects might be expected after administration of a potent antagonist [29]. A role for CXCR2 in neutrophil migration *in vivo* has been demonstrated in several animal models [22,30], but less is known of the *in vivo* functions of CXCR2 expression on structural cells or on other populations of inflammatory cells, such as dendritic cells, mast cells and T-lymphocytes, which also express CXCR2.

An interesting and unexpected finding of this study was the effect of AZD8309 on lung function following LPS challenge. As shown in Figure 4, the LPS-induced initial fall in FEV_1_ was similar for AZD8309 and placebo. However there appears to be a faster recovery after AZD8309 compared to placebo, which may reflect the effect of reducing neutrophil recruitment into the lungs and the reduced effects of mediators, such as reactive oxygen species and other bronchoconstrictor mediators, released from neutrophils on lung function.

This study details the use of a small number of healthy subjects in a short and simple challenge procedure which is reasonably reproducible. In order to validate our findings further it would be necessary to expand the trial to accommodate not only larger numbers patients COPD, severe asthma or CF. Furthermore, acute exacerbations of these pulmonary diseases are associated with increased neutrophilic inflammation in the airways so CXCR2 antagonists may be effective in preventing and treating exacerbations [31]. In addition, oral administration of an anti-inflammatory treatment has a significant advantage as it may improve access to peripheral airways compared to inhaled administration and this is particularly important in COPD and severe asthma.

## Conclusion

We have demonstrated that a CXCR2 antagonist given by oral administration can significantly inhibit both the inflammatory cellular response (neutrophils) and selected mediators in sputum following LPS challenge. There was a 47% reduction in macrophage numbers, although this did not achieve statistical significance. The LPS challenge model remains a useful technique for the evaluation of novel anti-inflammatory drugs in early stage development.

Our observations support the development of AZD8309 and other CXCR2 antagonists for the treatment of inflammatory pulmonary and non-pulmonary diseases characterized by neutrophilia.

## Abbreviations

ANOVA: Analysis of variance; b.i.d: Twice a day (bis in die); BMI: Body mass index; BSA: Bovine serum albumin; CF: Cystic fibrosis; CRP: C-reactive protein; Cmax: Maximum concentration; CXCR2: CXC chemokine receptor type 2; COPD: Chronic obstructive pulmonary disease; CV: Coefficient of variation; DTT: Dithiothreitol; ECG: Electrocardiogram; EDTA: Ethylenediaminetetraacetic acid; FEV1: Forced expiratory volume in 1 second; FVC: Forced vital capacity; GROα: Growth regulated oncogene alpha; IL-8: Interleukin-8; LPS: Lipopolysaccharide; LTB4: leukotriene B_4_. MCP-1; NE: Neutrophil Elastase; PBSA: Phosphate buffered saline plus 0.1% bovine serum albumin; PC20: Provocative concentration which produces a decrease in FEV_1_ by 20%; rpm: Rotations per minute; S.D: Standard deviation.

## Competing interests

B Leaker has received research funding from Astra Zeneca. PB has served on Scientific Advisory Boards of AstraZeneca, Boehringer-Ingelheim, Chiesi, Daiichi-Sankyo, GlaxoSmithKline, Novartis, Nycomed, Pfizer, Teva and UCB and has received research funding from Aquinox Pharmaceutiocals, AstraZeneca, Boehringer-Ingelheim, Chiesi, Daiichi-Sankyo, GlaxoSmithKline, Novartis, Nycomed, Pfizer and Prosonix. He is also a cofounder of RespiVert (now part of Johnson & Johnson) which has discovered novel inhaled anti-inflammatory treatments for asthma and COPD. B O’Connor has received research funding from Astra Zeneca.

## Authors’ contributions

BL Author, designed study, Investigator performed study, analysed data. PJB Co-author, designed study, analysed data. BO’C Investigator performed study. All authors read and approved the final manuscript.
